# The Role of Atoh1 in Mucous Cell Metaplasia

**DOI:** 10.1155/2012/438609

**Published:** 2012-02-14

**Authors:** Yoshihisa Nakamura, Yuki Hamajima, Masahiro Komori, Makoto Yokota, Motohiko Suzuki, Jizhen Lin

**Affiliations:** ^1^Department of Otolaryngology, Nagoya City University School of Medicine, 1 Kawasumi Mizuho-cho, Mizuho-ku, Nagoya, Aichi 467-8601, Japan; ^2^Department of Otolaryngology, Kochi University School of Medicine, 2-5-1 Akebono-cho, Kochi 780-8520, Japan; ^3^Department of Otolaryngology, University of Minnesota School of Medicine, 2001 6th Street SE, Minneapolis, MN 55455, USA

## Abstract

A key issue in otitis media is mucous cell metaplasia which is responsible for mucous hypersecretion and persistence of the disease. However, little is known about the molecular mechanisms of mucous cell metaplasia in otitis media. Numerous studies of intestinal epithelial homeostasis have shown that Atonal homolog 1 (Atoh1), a basic helix-loop-helix (bHLH) transcription factor, is essential for the intestinal goblet cell differentiation. On the other hand, SAM-pointed domain-containing Ets transcription factor (SPDEF), a member of the “Ets” transcription factor family, has been reported to trigger the mucous cell metaplasia of pulmonary infectious diseases or athsma. Recent studies have demonstrated the relation of these factors, that is, *Spdef* functions downstream of *Atoh1*. We could take the adventages of these findings for the study of otitis media because both middle ear and pulmonary epithelia belong to the same respiratory tract. Atoh1 and SPDEF could be the therapeutic targets for otitis media associated with mucous cell metaplasia which is frequently considered “intractable” in the clinical settings.

## 1. Mucous Cell Metaplasia in Intractable Otitis Media

 One of the most characteristic features of intractable otitis media is mucous hypersecretion. Chronic otitis media, caused by a prolonged bacterial infection, is characterized by ear discharge that is rich in mucus. Otitis media with mucoid effusion, common in young child otitis media patients and caused by a combination of bacterial infection and dysfunction of the Eustachian tube [1], is characterized by thick fluid accumulation in the middle ear cavity. Eosinophilic otitis media, which is a newly recognized intractable otitis media with bronchial asthma, is characterised by the presence of a highly viscous yellow effusion containing eosinophils [2]. 

The pathological basis of the mucous hypersecretion is mucous cell metaplasia, abnormal transformation of the normal respiratory tract mucosa into a thickened mucosa with a high density of the goblet cell population in response to microorganic infections or asthma. In a murine model of asthma, there was a dramatic increase in goblet cell number accompanied by a 75% decrease in Clara cells and a 25% decrease in ciliated cells after allergen challenge with a total number of epithelial cells being constant [3]. The increase of goblet cells and decrease of ciliated cells are also reported in the rat model of otitis media with effusion induced by the endotoxin plus Eustachian tube obstruction [4]. These findings suggest that the ciliated cells convert to goblet cells under the pathological conditions via a biologic process called cell transdifferentiation. It has been demonstrated that various materials such as bacteria [1], lipopolysaccharide [5], tumor necrosis factor-alpha (TNF-*α*) [6], and interleukin-10 (IL-10) [7] are involved in mucous cell metaplasia of the middle ear mucosa.

Mucins serve critical functions in the host defense against microorganisms. They are the principal structural components of the mucociliary transport system and the pathogens entrapped in a mucin gel layer are eliminated by constant beatings of the cilia on the mucosal surface. Under normal conditions, few mucous cells exist in the middle ear mucosa and they are mainly distributed in the orifice of the Eustachian tube, promontory area, and inferior tympanium. However, under pathological conditions inducing mucous cell metaplasia, an increased goblet cell population in the mucosa produces excessive mucins, while the loss of ciliated cells in the mucosa results in the dysfunction of the mucociliary transport system, leading to subsequent problems such as fluid accumulation and repeated infections. It is necessary to understand the disease mechanism of mucous cell metaplasia in order to cure this intractable illness.

## 2. The Role of Atoh1 in Mucous Cell Differentiation

*Atonal homologue 1 *(*Atoh1*), also called the *mouse atonal homolog 1 *(*Math1*) gene in mice and the *human atonal homolog 1 *(*Hath1*) gene in humans, is one of the candidate genes which play a crucial role in the mucous cell metaplasia. *Atoh1* is a basic helix-loop-helix (bHLH) transcription factor that is essential for the intestinal goblet cell differentiation. It has been shown that goblet cells are completely abrogated in the intestine of *Math1*-null mouse [8]. *Math1*-null embryos die at birth due to respiratory failure and lack of many specific cell lineages, including not only intestinal secretory cells but also cerebellar granule neurons, spinal cord interneurons, and inner ear hair cells [9].

**Figure 1:**
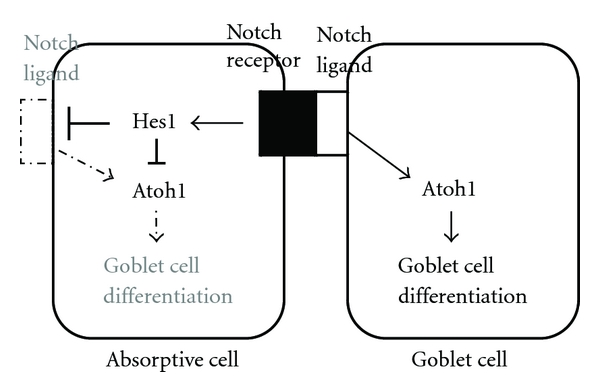
A schematic representation of lateral inhibition by a Notch ligand. A Notch ligand secreting cell releases a Notch ligand that binds to the Notch receptor of a neighboring cell. The action inhibits the activity of Atoh1 through Notch target gene Hes1 in the cell, leading to the formation of an absorptive cell. While a Notch ligand secreting cell has a high activity of Atoh1, leading to the differentiation of a goblet cell.

The role of Atho1 in goblet cell differentiation has been well studied in the intestine. The epithelium of the small intestine is composed of four different cell types: absorptive enterocytes and three secretory lineages consisting of mucus-secreting goblet cells, hormone-secreting enteroendocrine cells, and antimicrobial peptide-secreting Paneth cells. The intestinal epithelium is a highly dynamic tissue and is replaced by newly differentiated cells from multipotent stem cells residing near the base of the Crypts in every 2–7 day [9]. The differentiation for an absorptive or secretory cell linage is controlled by the Notch signaling system in a manner of lateral inhibition (Figure 1). Notch is a cell surface receptor that binds to Notch ligands, Dll (Delta-like), and Jagged families. Notch ligand, secreted from a progenitor cell which has a fate to become a goblet cell, binds to the Notch receptor on the other neighboring progenitor cell. This Notch receptor activation decides the fate of the neighboring cell because the Notch target gene *Hairy/enhancer-of-split 1 *(*Hes1*) inhibits the Atoh1. In this way, the neighboring progenitor becomes an absorptive cell. Akiyama et al. [10] has shown *Atoh1* is one of the downstream genes of Notch ligands and *Atoh1* is involved in the mechanisms of cellular differentiation from progenitor cells to goblet cells. They showed that Dll1 and Dll4 were selectively expressed in goblet cells within the human colonic epithelium and knockdown of Dll1 abrogated the *Hath1* and *MUC2* gene expression under activation of the goblet cell genes by a Notch signal inhibitor, suggesting the Notch ligand triggers the goblet cell differentiation through *Atoh1* activation. They have also reported forced expression of the Notch1 intracellular domain, an intracellular component of the Notch receptor, significantly suppressed *Dll1, Dll4, MUC2*, and *Hath1* gene expression, suggesting that notch receptor activation prevents *Atoh1* from triggering goblet cell differentiation by blocking the Notch ligand expression. 

 MUC2 is specifically expressed in the goblet cells of the intestine and thought as a goblet cell marker [11]. Park et al. found that Hath1 directly activated transcription of *MUC2* gene in the human intestinal epithelial cells [12]. In the human gastric cancer cells, overexpression of Math1 strongly enhanced both the MUC6 and MUC5AC mRNA transcript levels and knockdown of the *Hath1* gene significantly decreased the expression of both mucin genes [13].

The role of *Atoh1* in mucous cell development is well studied. However, the role of *Atoh1* in mucous cell metaplasia under diseased conditions is underinvestigated. An infection of whipworm (*Trichuris muris*) in the mouse intestine is reported to induce mucous cell metaplasia where *Math1* is significantly upregulated in the mRNA level [14].

## 3. The Role of SPDEF in Mucous Cell Metaplasia

 SAM Pointed Domain ETS Factor (SPDEF, also termed PDEF or PSE) is another candidate controlling mucous cell metaplasia. It is a member of the “Ets” family which regulates a number of biological processes, including cell proliferation, differentiation, and invasion. SPDEF was first described as a factor interacting with the androgen receptor to enhance expression of the prostate-specific antigen (PSA) promoter in vitro [15].

The role of SPDEF in mucous cell metaplasia is well attended in the study of lung diseases. SPDEF was markedly increased at sites of mucous cell metaplasia in bronchial tissues from patients with Cystic fibrosis or cigarette smoking [16]. In a murine model of asthma, the expression of SPDEF was also increased at sites of mucous cell metaplasia caused by IL-13 and dust mite allergen [17]. Chen et al. [16] have shown the Clara cell turns to goblet cell in the lung within 3 days after expression of SPDEF using a transgenic mouse model where the expression of *Spdef* gene under the Clara cell-specific promoter is controlled by doxycyline concentration (Scgb1a1-rtTA/TRE2-*Spdef*). This process is rapidly reversible and associated with the restoration of the Clara cells after stopping of the SPDEF expression. They have also reported some genes under the control of SPDEF. An mRNA microarray analysis study showed the expression of SPDEF influenced more than 300 genes including mucin 16 (*MUC16*), anterior gradient 2 (*AGR2*), glucosaminyl (*N-acetyl*) transferase 3, mucin type (*Gcnt3*), and chloride channel calcium activated 1 (*Clca1*). The involvement of these genes was confirmed by others. MUC5AC, AGR2, and Clca1 induced by IL-13 treatment were reduced in *Spdef* knockout human airway epithelial cells [18]. In colon cancer cells to activate the goblet cell genes by Notch signal inhibitors, knockdown of *Spdef* also repressed the expression of *MUC2* and *AGR2* [19]. Mucin is a large-molecular-weight glycoprotein and mucin production requires several steps including transcription of a *MUC* gene, holding, multimerization, and glycosylation. *MUC* genes code core mucin protein. *AGR2* is a member of protein disulfide isomerase (PDI) family which is critical for efficient formation of correctly arranged disulfide bonds in the endoplasmic reticulum (ER) [20]. *Gcnt3* is involved in the synthesis of a core structure in the mucin glycan chain. *Clca1*, whose mouse homolog is called as *Gob5*, is believed to have a role in secreting chloride anions into the lumen and contributing to the salt and water composition of secreted mucins [21]. All these genes contribute the production of mature mucins, which is conducted in the well-differentiated goblet cells. Inversely, blockage of SPDEF results in a failure of goblet cell maturation. The intestine of *Spdef* knockout mice revealed a severe loss of mature goblet cells and Paneth cells accompanied by accumulation of immature secretory progenitors. The typical aberrant goblet cells in *Spdef* knockout mice exhibit a clear brush border similar to adjacent enterocytes and carry poorly defined vacuoles in their cytoplasm. These immature goblet cells probably initiate the differentiation of goblet cells because they expressed trefoil factor 3 (TFF3), which is one of the goblet cell markers and thought to help in the oligomerization of mucin polysaccharides, although they did not have Alcian Blue Periodic Acid-Schiff (AB-PAS)-positive granules [22]. These data suggests SPDEF is a factor to serve for the terminal differentiation of goblet cells rather than to initiate or trigger the differentiation of goblet cells. SPDEF is thought to be a key transcription factor to start a series of sequential gene activations for the terminal differentiation and maturation of goblet cells.

## 4. *SPDEF* Functions as a Downstream Molecule of *Atoh1*

**Figure 2:**
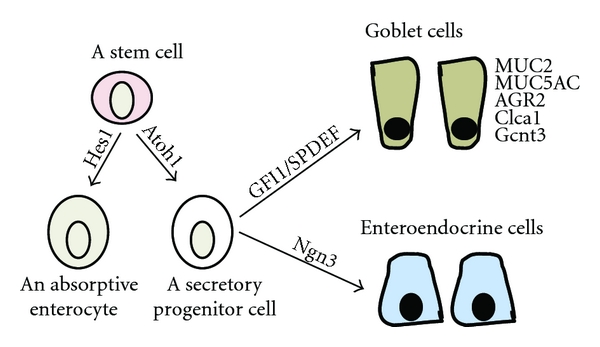
The differentiation path of a goblet cell starting from a progenitor and the genes involved in this process. An intestine stem cell becomes an absorptive cell in the absence of the Atoh1 protein but activation of Hes1 or becomes a secretory progenitor cell under the influence of Atoh1 protein with the potential to become either enteroendocrine cells under the direction of neurogenin 3 (Ngn3) or goblet cells under the direction of GFI1 and SPDEF.

 Accumulated pieces of evidence indicate that *Spdef* functions downstream of *Atoh1*, and this is mediated by another transcription factor, growth-factor-independent 1 (*GFI1*), which is normally expressed in the both Paneth and goblet cells (Figure 2). SPDEF was absent from intestine of the *Math1*-null mouse and was also quantitatively reduced in the *GFI1*-null mouse [19]. *GFI1* is thought to be downstream of *Atoh1* because the *Math1* expression was still observed in the *GFI1*-null mouse [9]. *Atoh1*-null crypts lack all the intestinal secretory cells. On the other hand, *GFI1*-null crypts lack Paneth cells, have few goblet and supernumerary enteroendocrine cells. These findings indicate the selection between secretory cells (goblet cells, Paneth cells, and enteroendocrine cells) and absorptive cell is dependent on *Atoh1*, and the selection between goblet/Paneth and enteroendocrine cells is dependent on *GFI1* [9].

## 5. The Role of *Atoh1* in Otitis Media

**Figure 3:**
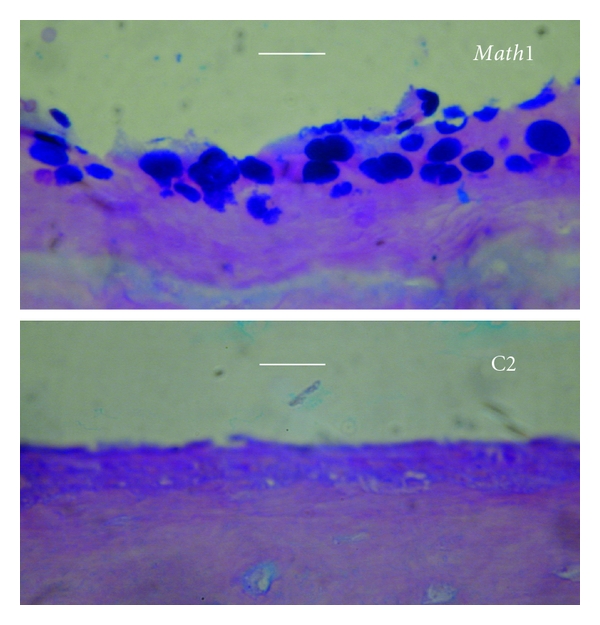
*Math1* transfection increases the mucous cells in the middle ear mucosa of mice. Full-length *Math1* cDNA was cloned into a protein-expressing vector (C2, pEGFP, Clontech). To study the role of *Math1* in the middle ear mucosa, bilateral bullae of 5 mice were transfected with 10 *μ*L of *Math1 *and empty vectors, respectively, at 1.4 *μ*g/mL in Opti-MEM containing Lipofectin at 6 *μ*g/mL via the tympanic membrane approach. Transfected animals were sacrified 7 days after *Math1* transfection for harvest of the bullae. *Math1* transfection had more AB-PAS positive cells in the mouse middle ear mucosa (upper) than empty vector (C2) transfection (lower). Bar = 10 *μ*m.

**Figure 4:**
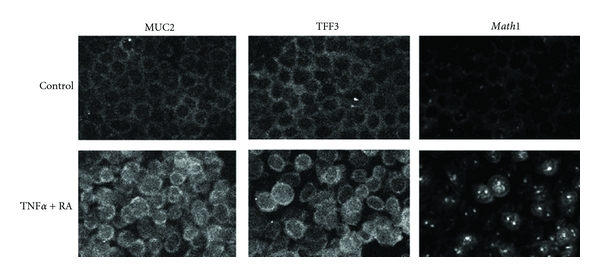
Mouse middle ear epithelial cells were incubated with 20 ng/mL of TNF*α* and 10^−9^ M of RA for two weeks on 8-well chamber slides (media and factors were supplied every two days). Immunohistochemistry showed that these factors increased the expression of goblet cell markers, MUC2 and TFF3, suggesting that this could be a model of mucous cell metaplasia in infectious otitis media. Math1 was located only in the cytoplasm in the control culture, however, the administration of TNF*α* + RA made Math1 translocate into nuclei. This Math1 activation (translocation to nuclei) probably triggers the expression of MUC2 and TFF3.

There is no report to discuss the involvement of *Atoh1*, *GFI1*, or *Spdef* in otitis media. However, the similarity of the middle ear epithelium to the pulmonary epithelium, *Atoh1*, the upstream gene of *Spdef* which is involved in several pulmonary diseases, may also have a role in the mucous cell metaplasia of otitis media. To test this hypothesis, we performed the transfection of *Math1* in the mouse middle ear. It was found that mucous cell numbers were significantly increased in *Math1* transfected middle ears compared with empty vector transfection (Figure 3). We also evaluated the effects of administration of TNF*α*, which is a common proinflammatory cytokine induced by bacterial or virus infection, and retinoic acid (RA) on mouse middle ear epithelial cells [23]. The administration of these factors for two weeks synergistically promoted the expression of MUC2 and TFF3 with an activation of Math1 in vitro (Figure 4). These data suggest that Atoh1 activated by proinflammatory cytokines triggers the mucous cell metaplasia in the middle ear epithelium as well.

## 6. Conclusions

 Over the past decade there have been significant advances in the studies of intestinal epithelial homeostasis and pulmonary mucous cell metaplasia. The former studies found Atoh1 as a factor to trigger the differentiation of goblet cells, and the latter studies found SPDEF as a factor to be responsible for mucous cell metaplasia, respectively. Now we can take advantages of these molecular understanding about mucous cells for the therapy of otitis media. Mucous cell metaplasia is a very primitive response to pathogens. Originally, this response is to protect the mucociliary system by secreting more mucins and their chaperones. These secreted substances are supposed to lubricate the lumen of the respiratory tract and discharge hazardous materials. However, mucous cell metaplasia also disturbs the well-organized mucociliary transport system at the expense of ciliated cells. Excessive mucus severely impairs the function of the mucociliary transport system and leads to subsequent infections. To make matter worse, bacterial cell wall components, or inflammatory cytokines trapped in accumulated mucus can be a driving force again for mucous cell metaplasia and thus form a vicious cycle. This vicious circle could explain why mucous cell metaplasia prolongs infectious diseases on a long-term basis. The effective blockage of the Atoh1, SPDEF, or GFI1 expression in the early phase of disease may be a new strategy for treating intractable otitis media. It could break the vicious circle of mucous cell metaplasia and lessen the burden of otitis media patients with mucoid effusion.
